# St. Louis Hospital Reports

**Published:** 1843-06

**Authors:** M. L. Linton

**Affiliations:** Professor of Obstetrics in the Medical Department of the University of St. Louis


### THE WESTERN JOURNAL OF MEDICINE AND SURGERY.

JUNE, 1843.

Art. I.—*St. Louis Hospital Reports. No.* 1. By M. L. Linton, M. D., Professor of Obstetrics in the Medical Department of the University of St. Louis.

I propose under the above caption to detail, in as concise a manner as possible, some of the more interesting surgical cases treated by Professor Brainard, of the Medical Department of the University of St. Louis. I proceed then directly to my task, and *first* to two cases of ununited fracture of about twelve weeks standing, treated by the starch bandage, *or immoveable apparatus.*

One was a fracture of the humerus just below the insertion of the *pectoralis major.* It had been treated by the ordinary method—wood splints, &c.—without success. A simple roller was now applied to the arm; four pieces of bookbinder's pasteboard moistened were placed over the roller, the *outer* piece of pasteboard extending to the tip of the shoulder. On these was spread a coating of dissolved starch; and another roller, imbued with the same, applied over the pasteboard. A simple roller binding the arm to the body (a thick compress having been placed between them) completed the dressing. I should remark, however, that two wooden splints were applied, to remain until the starch should dry, when they were removed—the apparatus becoming *so firm* as to deserve the epithet "*immoveable.*" At the end of seven weeks this apparatus was removed, when consolidation of the fracture and the form of the member were found to be perfect.

The *second* was a case of compound fracture of the tibia, accompanied by extensive laceration of the soft parts. This had been treated by the *fracture-box* until the *opening* and the injuries of the soft parts were healed. The immoveable apparatus was applied as before; two layers of the dry roller being applied next the skin. This case was not so fortunate. At the end of six days the member became painful; the apparatus was removed; and several ulcerations at the points of injury were observed. In his observations on these cases, the Professor remarked that they were well calculated to illustrate the advantages and disadvantages of the immoveable apparatus, about which so much had been lately said. In the first case, the skin being intact and the bone covered by soft parts, no ill effects occurred, and the cure was effected with a facility afforded by no other method. In the second the bone was but slightly covered with soft parts, and neither these nor the newly formed cicatrices were able to resist the pressure of the apparatus. It is recommended to leave openings in the apparatus corresponding to the ulcers, or to the openings in the soft parts in compound fractures; but even with this precaution it is difficult sometimes to prevent the formation of ulcers and the discharge of thin secretions beneath the bandage. Still the apparatus must not be blamed on account of *occasional* ill effects. When applied over recently formed tissues, or bony projections, these should be guarded by compresses of lint or cotton. But even with all these precautions bad effects *may* follow: thus the Professor had seen a case in the wards of Velpeau, in which a *simple* was converted into a *compound* fracture of the tibia, by the pressure of the extremity of the bone against the unyielding bandage; and death was the result.

Applied with caution and discrimination, it is invaluable in a great many cases—ununited fractures of the neck of the femur, or through the trochanters, are of this kind, whether the object be to effect a bony union or simply to allow the patient to walk about (on crutches) without great pain. The pieces of pasteboard in these cases should extend from the knee to the crest of the ileum, and the roller should be applied firmly around the pelvis as well as thigh. In a case of this kind, in which the patient was kept on his back until his health was so much impaired as to excite apprehensions for his safety, the apparatus thus applied enabled him in less than two weeks to walk about on crutches; the forces of the system were gradually restored.

In chronic cases the application of the damp rollers is liable to be followed by rheumatic inflammation. This should be guarded against by the sparing use of the starch, and by the use of warm applications until it is dried.

Case III.—*Varicose veins treated by needles and ligature.* The subject aged fifty-five years, had varicose veins of both legs attended by chronic ulcers. This case was of twenty-six years standing. The Lecturer remarked, that the propriety of attempting the radical cure of varicose veins had been called in question by many surgeons; it had also able advocates. This conflict of opinion had, he thought, arisen from the confounding of two different states of the veins. Varicose veins are produced by the obstruction of the venous canals to a *partial extent*, as is observed in pregnancy—varicose states thus induced, or by similar causes, are distinguished by a knotted appearance which arises from the hypertrophy of a portion of the circumference of the vessels; these enlargements appear first at the valves; increasing, the valves are forced and give way, the upper ones first, the lower ones as the disease advances. In the case before you, said the Professor, if you place your finger upon these enlarged branches below the knee, and then give a quick stroke upon the *saphena* where it dips down to join the femoral vein, you will feel the shock *below*, showing that the valves are imperfect. There is another class of enlarged veins, produced by the perfect obliteration of certain important venous trunks. The collateral veins enlarge to supply their place; these latter are hypertrophied in every sense of the term, and, when superficial, can be seen running in a serpentine or zigzag manner under the skin. He had seen an example of this vicarious enlargement in the service of Cruveilhier, in which, from obliteration of the ascending cava, the subcutaneous abdominal and thoracic veins were enormously enlarged; and another in private practice, in which the same result followed obliteration of the *saphena*. The difference between these kinds of cases is obvious: in the first, the circulation is retarded in the enlarged vessels; in the second, it is increased, these latter deserving the name of *suppleant* or vicarious veins. It would be bad practice to obliterate these; the former only call for an operation. But would it not be better to confine our efforts to palliatives? say the roller, laced stocking, and starch-bandage? The gravity of the disease and the great length of time required for such means to effect any thing, prevent us from hoping much from them. Let us examine then the different modes of obliteration. We pass over the ligature, excision, &c., as practised by the older surgeons, and come directly to the two favorite methods of the present day, viz: *the caustic* and *the needles with ligatures.* The latter of these methods is principally advocated by Velpeau. He passes a needle behind the vein and embraces it with a thread, as in the twisted suture, *so firmly as to occasion a sloughing* of the comprised vein and integuments. This usually causes but little pain or inconvenience. In twelve hundred cases operated in this way by Velpeau, but *one* death has occurred. The former method, practiced by Berard at the Neckar Hospital, consists in the production of the slough by the use of the potassa fusa, or the potassa cum calce. In both methods the same result is arrived at—the vein is obliterated. Berard already (eighteen months since) boasts of one hundred cases treated in this way, without one death. But, added the Professor, in visiting his wards I observed numerous cases of troublesome abscess, clearly the result of this mode of treatment. For this and other reasons I prefer the method of Velpeau. The Professor then operated as already indicated, placing the needles under the two principal trunks of the enlarged veins. At present, after four weeks, the veins are obliterated and no ill effects have followed.

January, 1843.

